# Heart rate at discharge in patients with acute decompensated heart failure is a predictor of mortality

**DOI:** 10.1186/s40001-020-00448-9

**Published:** 2020-10-08

**Authors:** Thomas Vollmert, Martin Hellmich, Natig Gassanov, Fikret Er, Seyrani Yücel, Erland Erdmann, Evren Caglayan

**Affiliations:** 1 Department III for Internal Medicine, University of Cologne, Cologne, Germany; 2 Institute of Medical Statistics and Computational Biology (IMSB), Faculty of Medicine and University Hospital Cologne, University of Cologne, Cologne, Germany; 3 Department II for Internal Medicine, Klinikum Idar-Oberstein, Idar-Oberstein, Germany; 4 Department of Cardiology and Electrophysiology, Klinikum Gütersloh, Gütersloh, Germany; 5 Department of Cardiology, University-Medicine Rostock, Rostock, Germany

**Keywords:** Heart rate at discharge, Acute decompensated heart failure, Mortality, HFrEF, HFmrEF

## Abstract

**Aims:**

Heart failure is a syndrome with increasing prevalence in concordance with the aging population and better survival rates from myocardial infarction. Morbidity and mortality are high in chronic heart failure patients, particularly in those with hospital admission for acute decompensation. Several risk stratification tools and score systems have been established to predict mortality in chronic heart failure patients. However, identification of patients at risk with easy obtainable clinical factors that can predict mortality in acute decompensated heart failure (ADHF) are needed to optimize the care-path.

**Methods and results:**

We retrospectively analyzed electronic medical records of 78 patients with HFrEF and HFmrEF who were hospitalized with ADHF in the Heart Center of the University Hospital Cologne in the year 2011 and discharged from the ward after successful treatment. 37.6 ± 16.4 months after index hospitalization 30 (38.5%) patients had died. This mortality rate correlated well with the calculated predicted survival with the Seattle Heart Failure Model (SHFM) for each individual patient. In our cohort, we identified elevated heart rate at discharge as an independent predictor for mortality (*p* = 0.016). The mean heart rate at discharge was lower in survived patients compared to patients who died (72.5 ± 11.9 vs. 79.1 ± 11.2 bpm. Heart rate of 77 bpm or higher was associated with an almost doubled mortality risk (*p* = 0.015). Heart rate elevation of 5 bpm was associated with an increase of mortality of 25% (*p* = 0.022).

**Conclusions:**

Patients hospitalized for ADHF seem to have a better prognosis, when heart rate at discharge is < 77 bpm. Heart rate at discharge is an easily obtainable biomarker for risk prediction of mortality in HFrEF and HFmrEF patients treated for acute cardiac decompensation. Taking into account this parameter could be useful for guiding treatment strategies in these high-risk patients. Prospective data for validation of this biomarker and specific intervention are needed.

## Background

Heart failure is a clinical syndrome with a poor prognosis and increasing prevalence [1]. Episodes with acute cardiac decompensation of chronic heart failure are associated with deterioration of left ventricular function and worsening of the clinical course [2]. An elevated resting heart rate is predictive for excessive morbidity and mortality for both men and women with and without cardiovascular diseases [3]. Multiple studies have confirmed this association for patients with coronary artery disease, acute myocardial infarction and heart failure [4–6].

The deleterious effects can be explained from a mechanistic point of view: an elevated heart rate leads to ischemia, abnormal calcium handling, accelerated atherosclerosis, and increased risk of plaque rupture [7]. Furthermore, an elevated resting heart rate is associated with cardiac risk factors like high systolic blood pressure, lack of physical activity, smoking and alcohol [3]. In addition, it indicates a poor cardiac function and decreased cardiorespiratory fitness.

In heart failure, an elevated heart rate preserves cardiac output in the setting of decreased stroke volume, however, this compensatory mechanism becomes maladaptive on the long term by increasing myocardial oxygen demand and reducing coronary perfusion time. Furthermore, persistent tachycardia becomes itself involved in the development of left ventricular dysfunction or heart failure, as seen in tachycardia induced cardiomyopathy [8].

In stable chronic heart failure, a slower resting heart rate is a valuable biomarker associated with a better outcome independent of left ventricular ejection fraction when patients are in sinus rhythm [9]. Therapeutically, the beneficial role of betablocker therapy in chronic heart failure patients has been addressed to reduction in heart rate, in addition to the reduction of the incidence of arrhythmias, sudden cardiac death and providing protection from ischemia [10]. Medical therapies targeting heart rate in sinus rhythm by blocking the I_*f*_ current in pacemaker cells have additionally been proven to be advantageous in reducing cardiovascular morbidity and mortality in HFrEF patients with a heart rate ≥ 70 bpm despite optimal medical therapy in prospective multi-center studies [11, 12]. The current ESC heart failure guidelines for heart failure therefore recommend heart rate reduction with the I_*f*_ channel blocker ivabradine for symptomatic patients with chronic systolic heart failure in sinus rhythm and a heart rate of ≥ 70 bpm as an adjunct therapeutic strategy after establishment of a treatment with a maximum tolerated dose of beta-blockers, ACE-I (or ARB) and a MRA [13]. Even a co-administration strategy of ivabradine and beta-blockers during hospital admission for acute decompensation in HFrEF patients seems to be beneficial by improving systolic function, as well as functional and clinical parameters at short term [14].

While the effect of elevated heart rate for prognosis and therapy of cardiovascular morbidity and mortality is evident in chronic heart failure, data in this regard in acute heart failure are scarce and controversial. In addition, in the clinical situation of atrial fibrillation, the association of slower heart rate and improved outcome is less clear [15]. Several studies analyzing the value of heart rate on cardiovascular outcome in the context of ADHF have obtained non-conclusive results [16–22].

In this single-center study, we sought to address the prognostic value of discharge heart rate on all-cause mortality and rehospitalization for heart failure in patients with ADHF and a reduced ejection fraction (HFrEF and HFmrEF). For this purpose, we retrospectively analyzed electronic medical records of all hospitalized patients on an all comer basis with the diagnosis of ADHF in the year 2011 who were subsequently discharged into the ambulatory care setting after successful treatment.

## Methods

### Data collection and variables definition

We conducted a retrospective observational study on patients with HFrEF (*n* = 58) and HFmrEF (*n* = 20) who were admitted with the diagnosis of ADHF between January 1st, 2011 and December 31st, 2011 to the Heart Center of the University of Cologne. Eligible patients for analysis were identified by screening all International Classification of Diseases codes for heart failure from electronic medical records of patients hospitalized in the department of cardiology within this time frame. Patients hospitalized with new or worsening heart failure or patients who developed significant heart failure symptoms such that HF was the primary discharge diagnosis were included. To avoid selection bias, discharge letters from individual patients were reviewed to confirm or exclude ADHF in agreement with Framingham criteria [23].

Included patients had to be older than 18 years of age, diagnosed with clinical signs and symptoms for acute decompensated heart failure, functionally classified to be in New York Heart Association class III or IV, had a left ventricular ejection fraction of < 50% on transthoracic echocardiography and hospitalized in the cardiology unit for at least 24 h. All patients had to be successfully treated with guideline recommended heart failure treatment and discharged home after recompensation therapy.

We excluded all patients who died during the index hospitalization period. Furthermore, patients were excluded with missing echocardiography and ECG, patients with an incomplete electronic medical record and any condition likely to preclude follow-up.

Electronic medical record data were obtained from the hospital information system ORBIS™ (Agfa HealthCare Corporation), which is used for every patient treated in the University Hospital Cologne. Available data included demographic and clinical characteristics, comorbidities, previous therapies and interventions, laboratory and non-laboratory tests, medications and vital signs. Left ventricular ejection fraction (LVEF) was estimated with transthoracic echocardiography using Simpson’s method according to current international recommendations at least once during the index hospitalization. Heart failure was categorized as being ischemic or non-ischemic in etiology depending on the presence or absence of co-existing significant coronary artery disease detected by angiography or history of previous myocardial infarction. Heart rhythm was electrocardiographically determined. Discharge heart rate and blood pressure was determined from routine measurements obtained by the responsible nurse on the ward once at the day of discharge in conformance with the local protocol for obtaining vital signs.

### Outcome measures

The primary outcome was all-cause mortality. We compared clinical and laboratory characteristics of patients who survived or died until the end of the observation period. In addition, we further analyzed hospitalization for cardiovascular causes and any cause during this period. Referring to established risk score models we focused on a set of clinical and demographic factors. All data were available from clinical follow-up of the patients in the outpatient setting and obtained by electronic medical record review using ORBIS™. Vital status was assessed from clinical follow-up in an outpatient setting in collaboration with the general practitioner/cardiologist or by direct contact with the patients to determine the overall mortality from heart failure as the primary end point of this study.

According to heart rate at discharge, patients were further evaluated in quartiles of heart rates: < 65, 66–75, 76–85 and > 86 bpm. For further analysis, we dichotomized patients into another two groups: ≥ 77 bpm and < 77 beats per minute.

In addition, for validation purposes, we compared our follow-up data on mortality with the prognostic data on survival of our patients obtained from the Seattle Heart Failure Model, a reliable risk score for chronic heart failure patients [24].

The study was conducted according to the guidelines of the local ethical committee and in conformity to the principles of the Declaration of Helsinki.

### Statistical analysis

Conventional descriptive statistics were used to summarize the data collected in this clinical study. For continuous variables the mean and standard deviation were used to present the data, for qualitative variables count and percentage. Kolmogorov–Smirnov test and Shapiro–Wilk test were performed to assess any deviation from normal distribution. Cox proportional hazards model were used to evaluate multivariable association with all-cause mortality. Qualitative variables were compared between groups using the chi-square test or the Fisher exact test, and quantitative variables were compared by Student’s t test. The log-rank test was used to evaluate differences in all-cause mortality. A p value less than 0.05 was an indicator for statistical significance. Calculations were done with the software SPSS Statistics (IBM Corp., Armonk, NY, USA).

## Results

### Study population

**Fig. 1:**
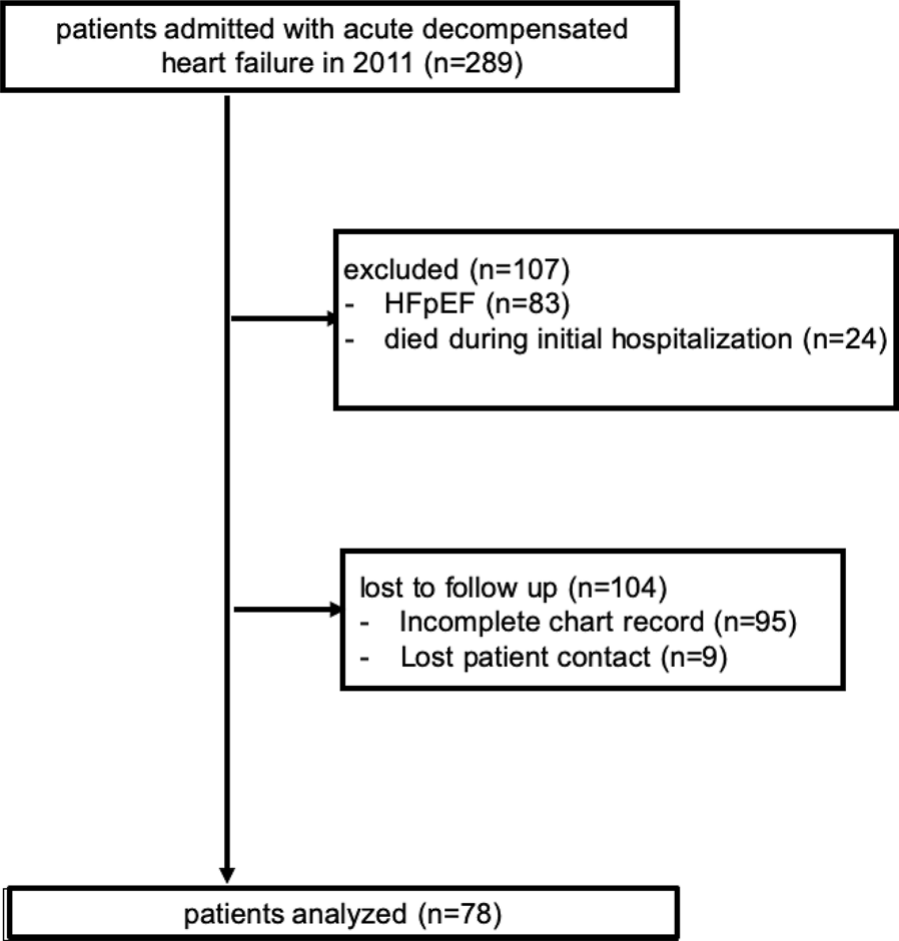
Study flow diagram

From January 1st, 2011 until December 31st, 2011, 289 patients were admitted with the diagnosis ADHF to the heart center of the University Hospital Cologne for further evaluation and medical treatment (Fig. 1). From these, *n* = 107 (37%) patients were excluded: *n* = 24 (8.3%) patients died during the index hospitalization period and n = 83 (28.7%) patients had heart failure with preserved ejection fraction (HFpEF) with an EF ≥ 50%. Furthermore, another *n* = 104 (36%) patients were lost-to-follow-up because of incomplete chart records (*n* = 95) and lost patient contact (*n* = 9).

### Clinical characteristics

The clinical characteristics of the final study population of 78 patients who had a complete clinical follow-up are presented in Table 1. The mean age was 69.4 ± 13.3 years. The majority (78%) of patients were male (vs. 22% female patients) and 66.7% of them were in functional class NYHA III (vs. 33.3% NYHA IV patients). The mean ejection fraction determined by echocardiography was 31.6 ± 9.6%. Heart failure was in 67.9% of ischemic origin and in 32.1% dilative. The mean resting heart rate at discharge was 75.0 ± 12.0 bpm [CI 72.3; 77.7 bpm] with a normal distribution.

**Table 1:** Baseline characteristics

Patient characteristics	all (*n*=78)	HFrEF (*n*=58)	HFmrEF (*n*=20)	*p* value
Age (years) ± SD	69.4±13.3	68.2±13.5	72.9±12.2	0.176
Body mass index kg/m2 ± SD	28.0±5.6	27.9±5.5	28.1±6.0	0.867
Male / female sex (%/%)	78 / 22	79/21	75/25	0.687
Ejection fraction (%) ± SD	31.6±9.6	27.3±6.9	44.0±3.2	<0.001
Mean heart rate (bpm) ± SD	75±12	76±13	72±10	0.184
Systolic blood pressure (mmHg)	115.7±18.5	111.8±15.7	126.9±21.8	0.001
NYHA functional class				
III/IV (%/%)	66.7/33.3	66/34	70/30	0.714
Heart failure subtype				
ICM/DCM (%/%)	67.9 / 32.1	66/34	75/25	0.433
Devices				
Pacemaker (n/%)	17/21.8	15/26.0	2/10.0	
ICD (n/%)	18/23.0	16/28.0	2/10.0	
CRT (n/%)	2/2.6	2/3.4	0/0	
Comorbidities				
Atrial fibrillation (n/%)	34/43.6	23/39.7	11/55.0	0.142
Diabetes mellitus (n/%)	33/42.3	24/41.4	9/45.0	0.926
Arterial Hypertension (n/%)	59/75.6	43/74.1	16/80.0	0.598
Hyperlipoproteinemia (n/%)	45/57.7	35/60.3	10/50	0.419
Chronic renal failure (GFR≤ 60 ml/min) (n/%)	16/20.5	14/24.1	2/10	0.070
Chronic obstructive pulmonary disease (n/%)	18/23.1	12/20.7	6/30.0	0.394
Current smoker (n/%)	30/38.5	23/39.7	7/35.0	0.904
Laboratory values				
Haemoglobin (g/dl) ± SD	13.2±1.9	13.3±1.9	12.8±2.0	0.295
Serum sodium (mmol/l) ± SD	138.9±4.1	139.0±4.1	138.7±4.6	0.734
Cholesterine (mg/dl) ± SD	162.9±41.7	165.2±45.1	156.3±40.0	0.432
Serum creatinine (mg/dl) ± SD	1.45±0.8	1.45±0.7	1.44±1.2	0.948
Blood urea nitrogen (mg/dl) ± SD	72.7±41.2	73.0±41.0	71.8±42.7	0.909
NTpro-BNP (pg/ml)	6823±9824	7285±10024	352±0	0.516
Medication				
ACE inhibitor or ARB (%)	82.1	84.5	75	0.669
Beta‐blocker (%)	91	91.4	90	0.813
Aldosterone antagonist (%)	65.4	72.4	45	0.120
Calcium antagonists (%)	10.3	3.4	30	0.030
Diuretics (%)	91	96.6	75	0.117
Digitalis glycosides (%)	28.2	29.3	25	0.200
Amiodarone (%)	9	12.1	0	0.103
Anticoagulant therapy (%)	33.3	31	40	0.220
Antiplatelet therapy (%)	48.7	44.8	60	0.192
Lipid lowering therapy (%)	50	50	50	0.762
Xantine oxidase inihibitors (%)	21.8	24.1	15	0.520

All patients received guideline directed medical therapy at discharge to the maximally tolerated dosage. ACE inhibitors or Angiotensin Receptor Blockers were taken by 84.6%, beta-blockers by 92.3% and mineralocorticoid receptor antagonists even by 65.4% of the study cohort.

23% of patients had an ICD as primary or secondary prevention, but only 3% had cardiac resynchronization therapy. The most prevalent comorbidity was arterial hypertension (75.6%), followed by hyperlipoproteinemia (57.7%), atrial fibrillation (43.6%) and diabetes mellitus (42.3%). Chronic renal failure with an estimated GFR of less than 60 ml/min was evident in 20.5% of all studied patients. Smoking habits were common with 38.5% current smokers. The mean NT-proBNP was 6823 ± 9824 ng/L.

We further performed an analysis on the different characteristics of HFrEF and HFmrEF patients in the study. HFmrEF patients had a significantly higher blood pressure at discharge and were significantly taking more calcium antagonists in their medication.

### Predictors of mortality

After a mean follow-up of 37.6 ± 16.4 months, 30 (38.5%) patients died (Table 2). Their mean survival time was 1.87 years [CI 1.46; 2.27 years]. Heart rate at discharge was a significant predictor of mortality (*p = *0.016, Pearson* r* = 0.28), besides known prognostic parameters like systolic blood pressure (*p = *0.004), NYHA functional class IV (*p* = 0.048), hemoglobin (*p* = 0.009), serum creatinine (*p = *0.002) and blood urea nitrogen (*p* < 0.001). Medication of xanthine oxidase inhibitors was associated with elevated mortality (*P* = 0.015).

**Table 2:** Characteristics of survivors vs. deaths

Patient characteristics	All (*n*=78)	Alive (n=48/62%)	*p* value	HFrEF (*n*=58)	Alive (*n*=34/59%)	*p* value	HFmrEF (*n*=20)	Alive (*n*=14/70%)	*p* value
	Dead (*n*=30/38%)			Dead (*n*=24/41%)			Dead (*n*=6/30%)		
Age (years) ± SD	70.8±13.7	68.6±13.1	0.472	68.7±14.2	67.9±13.2	0.826	79.2±7.1	70.2±13.1	0.136
Body mass index kg/m2 ± SD	26.7±6.6	28.7±4.7	0.13	27.7±6.8	28.1±4.4	0.809	23.1±4.7	30.3±5.2	0.009
Male/female sex (%/%)	77 / 23	79 / 21	0.79	83/17	76/24	0.525	50/50	86/14	0.091
Ejection fraction (%) ± SD	29.5±10.8	32.9±8.6	0.127	25.5±8.0	28.5±5.9	0.101	45.3±3.0	43.4±3.2	0.231
Mean heart rate (bpm) ± SD	79.1±11.2	72.5±11.9	0.016	80.1±11.7	73.3±12.7	0.041	75.2±8.9	70.6±9.8	0.339
Systolic blood pressure (mmhg)	108.2±16.3	120.4±18.4	0.004	108.5±16.7	114.2±14.7	0.173	107.2±16.1	135.4±18.3	0.004
NYHA functional class									
III/IV (%)	53/47	75/25	0.048	50/50	76/24	0.037	67/33	71/29	0.831
Comorbidities									
Atrial fibrillation (%)	57	35	0.107	46	35	0.419	100	36	0.014
Diabetes mellitus (%)	53	35	0.055	54	32	0.071	50	57	0.621
Arterial hypertension (%)	73	77	0.707	71	76	0.629	83	79	0.807
Hyperlipoproteinemia (%)	57	58	0.885	58	62	0.792	50	50	1.000
Chronic renal failure (gfr≤ 60 ml/min)	33	13	0.05	38	15	0.069	17	7	0.515
Chronic obstructive pulmonary disease (%)	30	19	0.251	25	18	0.496	50	21	0.201
Current smoker	33	42	0.728	38	41	0.94	17	50	0.362
Laboratory values									
Haemoglobin (g/dl) ± SD	12.5±2.1	13.7±1.7	0.009	12.8±2.0	13.8±1.7	0.060	11.4±1.9	13.5±1.8	0.027
Serum sodium (mmol/l) ± SD	138.0±4.9	139.4±3.4	0.125	138.5±4.4	139.3±3.8	0.429	136.0±6.7	139.7±2.4	0.239
Cholesterine (mg/dl) ± SD	155.4±38.3	167.5±46.0	0.231	159.7±40.4	169.0±49.4	0.441	138.3±23.7	163.9±41.0	0.174
Serum creatinine (mg/dl) ± SD	1.82±1.19	1.22±0.38	0.002	1.77±0.86	1.23±0.37	0.008	2.03±2.18	1.19±0.43	0.390
Blood urea nitrogen (mg/dl) ± SD	93.9±54.1	59.4±22.4	<0.001	92.5±54.6	59.2±19.0	0.008	99.7±57.0	59.8±30.1	0.053
Ntpro-bnp	11731±15582	6823±9824	0.18	11731±15582	4815±4760	0.23	n.a.	352	n.a.
Medication									
Ace inhibitor or ARB (%)	70	90	0.13	79	88	0.166	33	93	0.122
Betablocker (%)	90	92	0.664	92	91	0.738	83	93	0.281
Aldosterone antagonist (%)	57	71	0.374	58	82	0.281	50	43	0.419
Calcium antagonists (%)	0	17	0.062	0	6	0.481	0	43	0.159
Diuretics (%)	90	92	0.506	96	97	0.456	67	79	0.614
Digitalis glycoside (%)	33	25	0.458	33	26	0.496	33	21	0.414
Amiodarone (%)	13	6	0.287	17	9	0.366	0	0	n.a.
Lipid lowering therapy (%)	53	48	0.652	50	50	1	67	43	0.11
Xantine oxidase inihibitors (%)	40	10	0.015	46	9	0.013	17	14	0.203

Besides these significant variables, we could detect a tendency in terms of clinical and laboratory features that patients who survived were younger in age with a higher, slightly overweight BMI, with a better ejection fraction and renal function and less often have atrial fibrillation and diabetes mellitus as comorbidities.

When HFrEF and HFmrEF patients were compared separately, heart rate, NYHA class, serum creatinine, BUN and intake of xanthine oxidase inhibitors were significantly predictive for mortality in HFrEF patients, whereas BMI, systolic blood pressure, the presence of atrial fibrillation and low hemoglobin levels were predictive for mortality in HFmrEF patients.

### The role of heart rate at discharge

The mean heart rate of patients who survived was 72.5 ± 11.9 bpm [CI 69.0; 75.9 bpm] compared to 79.1 ± 11.2 bpm [CI 74.9; 83.3 bpm] of patients who died during the study period. Furthermore, heart rate at discharge correlated significantly with mortality (*r* = 0.28, *p = *0.013). Looking at the quartiles of heart rates at discharge, we observed an increasing mortality with higher heart rates at discharge. The 4 year mortality rates for the quartiles analyzed were as follows: < 65 bpm: 16.7%, 66–75 bpm: 40.9%, 76–85 bpm: 42.3% and > 86 bpm: 58.3%.

**Fig. 2:**
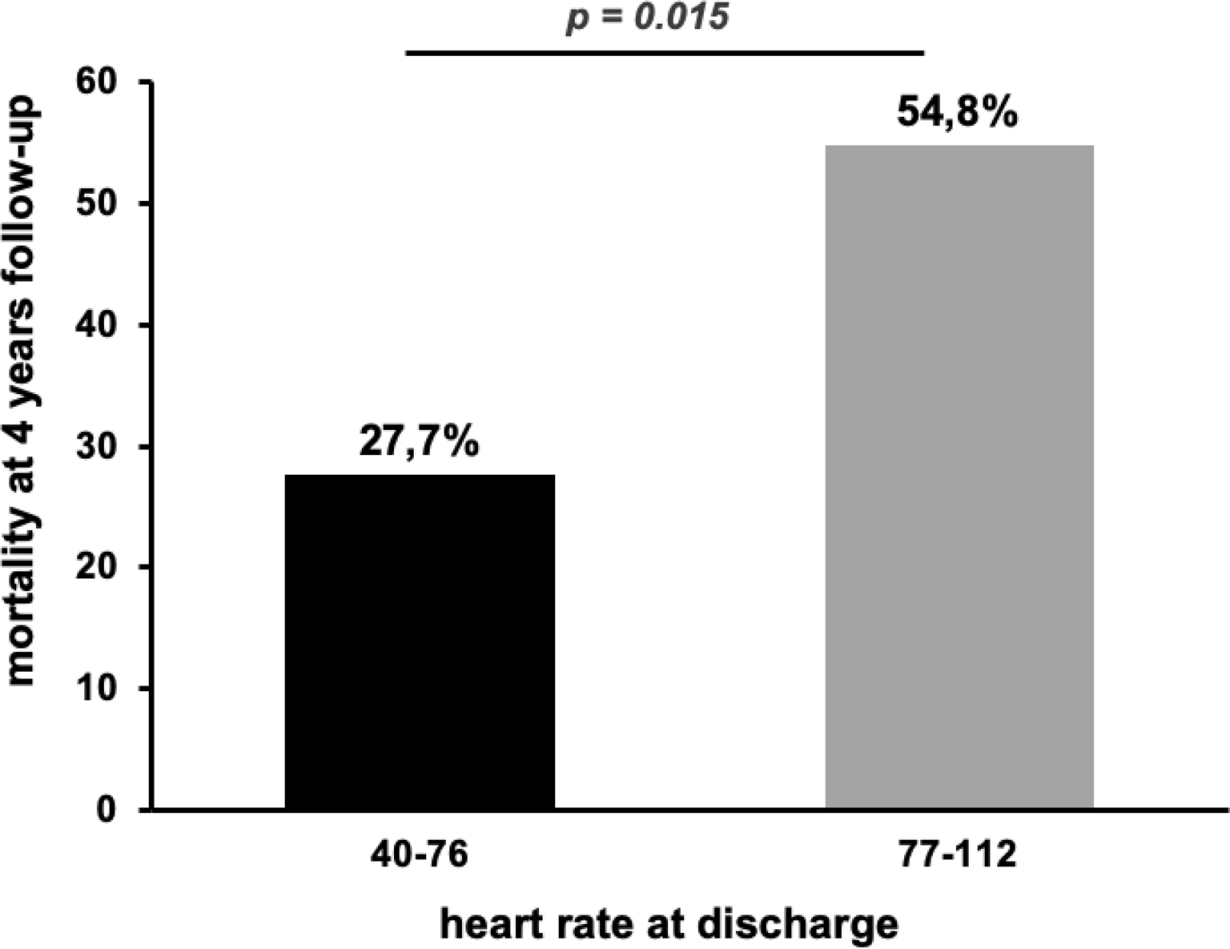
Mortality rates at 4 years follow-up in patients dichotomized to heart rates 40–76 and 77–120 bpm

**Fig. 3:**
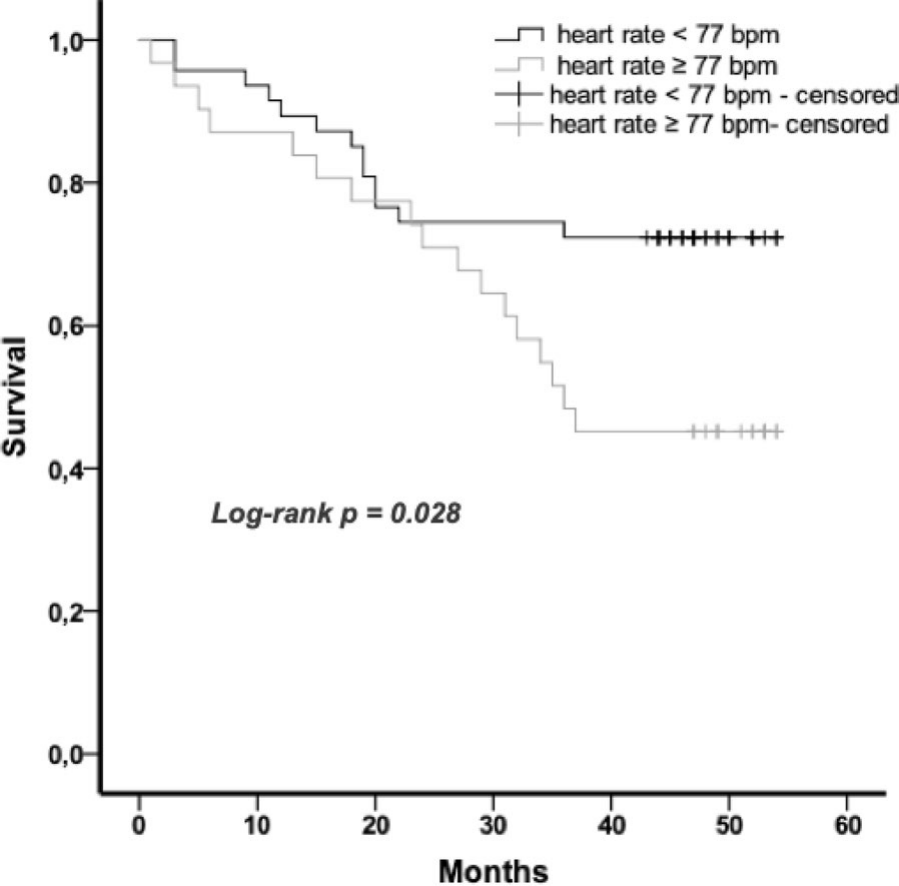
Kaplan–Meier survival curves of patients with a discharge heart rate ≥ 77 and < 77 bpm

Mortality rate at 4 years follow-up was nearly doubled (54.8% vs. 27.7%), when patients were dichotomized to a heart rate at discharge of greater or equal than 77–112 bpm compared to patients with a heart rate of 40–76 bpm (Fig. 2). This is also shown in the survival analysis using Kaplan–Meier estimate with a significantly better survival of patients with a heart rate at discharge of ≥ 77 bpm (*p* = 0.028) (Fig. 3).

We observed a linear relationship between mortality and heart rate at discharge with increasing odds for death by 5% with every heart beat per minute rising from baseline rate (*p* = 0.022).

We could not detect a significant correlation between discharge heart rate and readmission for heart failure or any other cause. Furthermore, we could not detect significant differences in the prediction of mortality due to heart rate at discharge whether patients were in sinus rhythm or had atrial fibrillation. Although patients with atrial fibrillation tended to have a worse prognosis per se (Table 2).

**Fig. 4:**
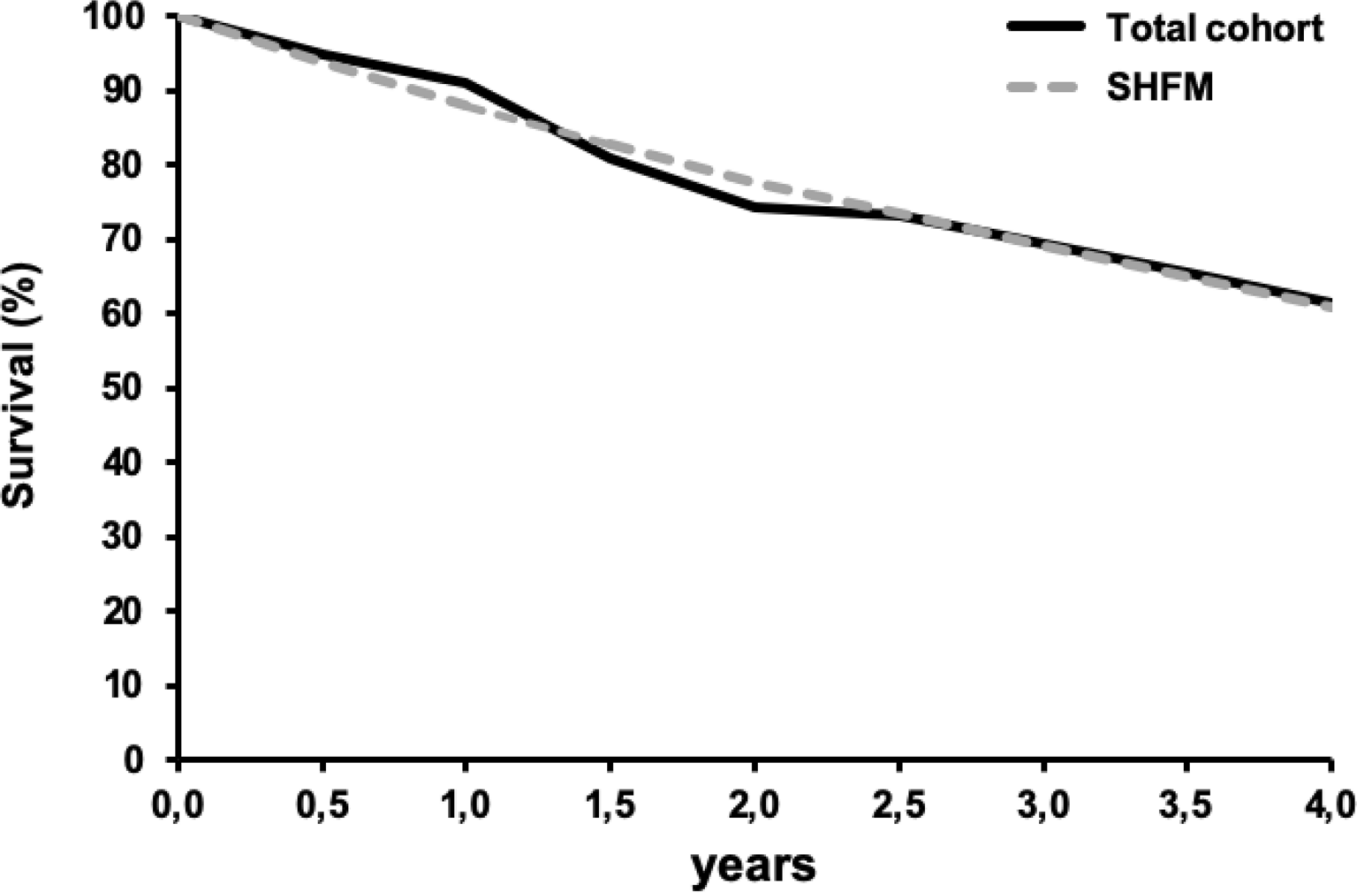
Kaplan–Meier actual survival curves of the study cohort compared with the survival curves calculated with the SHFM

Finally, to exclude bias due to hospital factors, we calculated the survival of each individual patient using the Seattle Heart Failure Model (SHFM) with the individual patient data available at discharge and compared the predicted survival curve to the actual survival of the cohort. Hereby, we could confirm that the effective survival curve was similar to the predicted by the SHFM, suggesting that the calculated factors in the investigated cohort were representative to a similar sick cohort in a bigger heart failure population (Fig. 4).

## Discussion

In this clinical investigation, we were able to demonstrate that heart rate at discharge is a predictor of mortality in patients with HFrEF and HFmrEF discharged to ambulatory care after an episode with ADHF. A heart rate ≥ 77 bpm was associated with a nearly two-fold increased mortality in this patient population. We could not detect differences for the role of this parameter in regard to the existing cardiac rhythm at discharge. In addition, this parameter was not predictive for rehospitalization.

The prognostic impact of heart rate in ADHF is still a matter of debate. In contrast to the predictive role of this biomarker in chronic systolic heart failure the role of heart rate in ADHF is much more controversial. This is partly due to differences in the time point when heart rate was measured during an acute decompensation period and focusing on different end points like in-hospital mortality and readmission in various studies [16–22].

Risk of in-hospital mortality and particularly mortality and rehospitalization for patients hospitalized with ADHF remains high [25]. It is increased in patients with impaired metabolic status, neurohormonal activation and reduced cardiac performance, gauged by BUN, serum albumin and cholesterol levels, systolic blood pressure, heart rate, and respiratory rate [26]. Particularly, admission heart rate has been shown to be an independent risk factor for mortality during the acute phase as well as in the long term. A higher heart rate on admission was independently associated in a J-shape relationship with higher in-hospital mortality in ADHF patients with the lowest mortality seen at heart rates of 70–75 bpm [16]. Furthermore, a higher heart rate at presentation in the emergency department with ADHF was associated with an increased 7 day mortality [27]. On the other hand, higher admission heart rate can also predict survival advantage in acute HF and improve left ventricular reverse remodeling [19, 28]. However, lower heart rate is also a marker for increased in-hospital mortality in ADHF, suggesting the existence of an ideal heart rate window in these patients [18].

Some studies have correlated the difference between admission and discharge heart rate to hard cardiovascular end points [20]. Patients presenting with tachycardia and discharged with a controlled heart rate were shown to have a better outcome than those admitted non-tachycardic or discharged with a non-controlled heart rate [19]. These observations are explained by the hypothesis that elevated heart rate in the initial period of ADHF may be an indicator of preserved cardiac reserve and chronotropic competence, as the ability of the cardiovascular system to respond to this extraordinary stress situation with an adrenergic burst is preserved. Hence, heart rate recovery indicates a functioning vagal arm of the autonomous nervous system resulting in a better prognosis in patients with systolic heart failure. Nevertheless, both admission heart rate as well as heart rate difference (admission–discharge heart rate) are complicated to introduce into routine clinic algorithms for risk prediction in ADHF.

A variety of short- and long-term mortality predictors have been analyzed in patients with ADHF. Nevertheless, application of a sophisticated risk-prediction algorithm to identify patients at high risk for mortality who might benefit from aggressive monitoring and intervention using various variables suggested in the Organized Program to Initiate Lifesaving Treatment In Hospitalized Patients With Heart Failure (OPTIMIZE-HF) trial are even more cumbersome to perform in routine clinical practice [17, 25, 26].

Only few studies like ours have concentrated on heart rate at discharge for risk prediction. This parameter has several advantages: it is easy and reliably to determine, routinely available and reflects the most stable condition the patient is able to achieve and therefore may be much more relevant for the future course. Indeed, an elevated discharge heart rate is independently associated with a poor prognosis in patients revascularized with percutaneous coronary intervention for stable angina or acute coronary syndromes as well as after acute myocardial infarction [21, 29].

Our results are in line with a big retrospective cohort study of registry data showing that a higher discharge heart rate after treatment for ADHF in unselected heart failure patients is associated with an increased risk of death and rehospitalization with an even higher risk in the first 30 days after discharge [30].

The strength of our study is a high percentage of patients with guideline-based medical and device therapy and the unique long-term follow-up period with a mean duration of more than 3 years. Besides that, compared to previous studies we focused on patients with primarily systolic heart failure with an ejection fraction < 50%. The population analyzed ended up with a sicker cohort of HFrEF and HFmrEF patients with a more severely reduced mean EF. Probably due to the nearly optimal guideline directed medical therapy and the younger mean age of our study cohort, the mortality rates were noticeably lower compared to previously published studies. However, observed survival rates similar to predicted survival rates calculated with the SHFM were confirmative to exclude selection bias.

Particular differences in the study cohort might account for the differences observed in the mortality rates. In contrast to our study, the population investigated by Laskey et al. included patients who were nearly a decade older (median age 80 years) with systolic and diastolic heart failure. They had a more preserved LVEF (median 45%). In addition, this cohort included fewer ICD patients, more women and less patients with heart failure due to ischemic origin. Lastly, the follow-up period in this analysis was only 12 months. Overall, both studies underscore the positive association between discharge heart rate and mortality in patients with heart failure.

While this association is true for patients in sinus rhythm, the data in regard to Afib patients are less clear. Atrial fibrillation is not only independently associated with adverse prognosis in chronic but also in acute heart failure at least up to 1 year post-discharge [31]. Only few studies have analyzed the relationship between heart rate and mortality in ADHF patients with atrial fibrillation thereby obtaining divergent results [22, 30]. In our study, we could not detect a difference in the association of heart rate at discharge and mortality between patients presenting in SR versus those with Afib, although there was tendency that Afib was more prevalent in patients who died (*p* = 0.107; Table 2). A recent meta-analysis of randomized controlled trials suggests that in regard to mortality a lower heart rate in stable HFrEF patients is associated with a better prognosis only when patients were in sinus rhythm, while this association was not seen in Afib patients [32].

The optimum heart rate at discharge in respect to mortality risk for heart failure patients is not clear. We performed an exploratory analysis of the heart rate–mortality association to determine a cut-off heart rate with incremental hazard. From previous and our investigation there seems to be an upper cut-off window of 75–76 bpm. When the resting heart rate is above this rate, mortality seems to increase disproportionately. This upper cut-off window might be higher in Afib patients, although we were not able to detect significant differences in our study due to the small sample size and supposedly higher heart rate variability during Afib.

Lastly, in regard to the observed significantly higher mortality in patients taking xanthine oxidase inhibitors, indeed, in a recent systematic review and meta-analysis of uric acid-lowering agents on cardiovascular outcome in patients with heart failure treatments, allopurinol treatment was associated with a significant increase in the risk for all-cause and cardiovascular mortality [33]. Whether the observed effect of an increased mortality in patients treated with a xanthine oxidase inhibitor in our study is due to a treatment effect or elevated uric acid levels leading to medical treatment characterizes a sicker heart failure population cannot be answered with the data presented. Xanthine oxidase inhibition with allopurinol has not been demonstrated to show additional benefit nor harm in high-risk HFrEF patients with elevated uric acid levels [34].

## Limitations

There are a few limitations of this study. This is a single-center retrospective analysis without having access to structured registry data. Hospital factors and a rather high number of patients who had to be excluded due to incomplete chart record might have influenced the results. In addition, we had to rely on the accuracy of documentation. It cannot be fully ruled out that physiologic variables may not have been accurately recorded or been subjected to rounding which could have influenced the results. Furthermore, not all possible prognostic parameters were available in the dataset. Residual measured and unmeasured confounding factors may influence the results. Nevertheless, a considerable number of events were analyzed and we tried to exclude bias considering every patient with the diagnosis of ADHF in our study.

## Conclusions

Taking into account heart rate at discharge in patients with ADHF and systolic heart failure may allow for greater discrimination of the highest risk patients. Further investigation should address whether heart rate reduction could be a therapeutic strategy in ADHF.

## Data Availability

All original data supporting the findings of the study are available from the corresponding author on request.
